# Influence of Different Sterilization Methods on the Surface Chemistry and Electrochemical Behavior of Biomedical Alloys

**DOI:** 10.3390/bioengineering10070749

**Published:** 2023-06-22

**Authors:** Anna Igual-Munoz, Jean-Ludovic Genilloud, Brigitte M. Jolles, Stefano Mischler

**Affiliations:** 1 Tribology and Interfacial Chemistry Group, Institut des Matériaux, Ecole Polytechnique Fédérale de Lausanne, CH-1015 Lausanne, Switzerland; jeanludovic.genilloud@gmail.com (J.-L.G.); stefano.mischler@epfl.ch (S.M.); 2 Swiss BioMotion Laboratory, Lausanne University Hospital and University of Lausanne (CHUV-UNIL), CH-1011 Lausanne, Switzerland; brigitte.jolles-haeberli@chuv.ch; 3 Institute of Microengineering, Ecole Polytechnique Fédérale Lausanne (EPFL), CH-1015 Lausanne, Switzerland

**Keywords:** sterilization, biomedical alloys, corrosion

## Abstract

Sterilization is a prerequisite for biomedical devices before contacting the human body. It guarantees the lack of infection by eliminating microorganisms (i.e., bacteria, spores and fungi). It constitutes the last fabrication process of a biomedical device. The aim of this paper is to understand the effect of different sterilization methods (ethanol-EtOH, autoclave-AC, autoclave + ultraviolet radiation-ACUV and gamma irradiation-G) on the surface chemistry and electrochemical reactivity (with special attention on the kinetics of the oxygen reduction reaction) of CoCrMo and titanium biomedical alloys used as prosthetic materials. To do that, electrochemical measurements (open circuit potential, polarization resistance, cathodic potentiodynamic polarization and electrochemical impedance spectroscopy) and surface analyses (Auger Electron Spectroscopy) of the sterilized surfaces were carried out. The obtained results show that the effect of sterilization on the corrosion behavior of biomedical alloys is material-dependent: for CoCrMo alloys, autoclave treatment increases the thickness and the chromium content of the passive film increasing its corrosion resistance compared to simple sterilization in EtOH, while in titanium and its alloys, autoclave and UV-light accelerates its corrosion rate by accelerating the kinetics of oxygen reduction.

## 1. Introduction

Biomedical implants and, in general, medical devices, must be sterile before implantation and contact with the human body. To do that, different physical and chemical techniques are available nowadays, and their use depends on the material properties and the final application of the device. Among them, gamma irradiation (G), steam autoclave (AC), ultraviolet irradiation (UV), dry heat and ethylene oxide (EO) are widely used in the biomedical field. Each of these different sterilization methods have their own advantages and limits [1] with more (gamma radiation) or less (such as autoclave) penetrability, very good compatibility with all materials (such as ethylene oxide) or low cost (such as autoclave). A very simple method is the use of 70% ethanol in water, which can also be considered a sterilization method.

Whatever the method, the sterilization process in the manufacturing of biomedical devices constitutes a crucial step to prevent infections of the implanted elements. It is the process that facilitates eliminating and stopping the production of microorganisms, such as bacteria, spores and fungi, and it is considered the last step of the surface modification before the device is in contact with the human body. Its importance is related to the fact that sterilization has to remove the maximum number of microorganisms without compromising key surface properties that may influence their interaction with the surrounding tissue (i.e., electrochemical reactivity).

The biocompatibility of implants is related to the implant–body interactions and, therefore, to the surface reactivity with the surrounding biofluids (corrosion). Since sterilization is the final step in the manufacturing process of implants, characterizing the influence of that process on the electrochemical behavior of the resulting surface is needed to understand the final implant performance in vivo. The importance of understanding the corrosion degradation mechanisms of biomedical implants is related to its consequences on metal ion release into the human body and other clinical implications, such as inflammation [2,3,4].

It has been previously reported for titanium and its alloys that cell adhesion and proliferation and bacterial attachment, which are directly related to the biocompatibility of those materials, are highly dependent on surface chemistry, crystallinity, roughness and wettability, which can be modified by the final sterilization procedure [5,6,7,8]. Indeed, sterilization by autoclaving has been shown to decrease the hydrophilicity of titanium surfaces due to the introduction of hydrophobic contaminants on the surface [9,10]. Autoclaving has also been shown to destroy nanotubular morphology during the sterilization of TiO_2_ nanotubes used for biomedical devices, while other methods, such as UV-light or plasma sterilization, did not cause any damage. However, all sterilization techniques influenced the cytocompatibility of some nanotubular structures [11]. Similar results were also found by Oh et al. [12], who also observed that dry or wet autoclaving generated different cell adhesion properties on TiO_2_ nanotubes due to the difference in air entrapment among the different autoclaving conditions, which modified the protein deposition in and on the nanotubes necessary for cell adhesion contacts.

With respect to the influence of bacterial growth depending on the sterilization process, Kumer et al. [13] observed that UV- and EtOH-sterilized samples decreased the bacterial growth on anodized TiO_2_ nanotubes, while autoclave sterilized samples showed the highest amount of bacterial growth. This effect was found to be dependent on the length of the nanotubes, with the 20 nm TiO_2_ structure showing the lowest bacterial growth.

Clearly, the final sterilization process modifies the surface properties of the treated implant, although very scarce literature [14] exists on its effect on the corrosion behavior. In this previous work, Thierry et al. [14] tested an electropolished NiTi biomedical alloy after sterilization using several procedures (ethylene oxide, steam autoclaved, paracetic acid and hydrogen peroxide plasma). The main influence of sterilization on the corrosion behavior of the NiTi was observed after steam autoclaving. NiTi showed an increase in corrosion potential and a decrease in localized corrosion resistance, which were related, by the authors, to the generation of surface defects during that sterilization process. However, no clear conclusions on which sterilization parameter was responsible for the different corrosion behavior were derived from this study.

Recently, an in vivo corrosion study testing pure titanium in synovial fluid directly extracted from patients has shown that the cathodic reaction of dissolved oxygen plays a key role determining the corrosion rate of the metal [15]. The cathodic reduction of oxygen is an electrochemical parameter that is significantly affected by the surface state of the metal. It is thus expected that the sterilization process, by modifying the metal surface, affects the corrosion of titanium and by extension biomedical alloys in general. 

The aim of this work is to characterize the effect of sterilization processes on the electrochemical behavior of common biomedical alloys used in orthopedic implants. The investigated materials are a low carbon CoCrMo alloy, a Ti6Al4V alloy and pure titanium. The latter was investigated in an anodized and non-anodized state to check for possible effects of TiO_2_ thick surface films. Samples were exposed to different sterilization processes: 70% ethanol/water, steam autoclave, UV-light and gamma-irradiation. The corrosion tests were carried out in a simulated body fluid. Corrosion was characterized by electrochemical methods with a particular emphasis on the cathodic kinetics (oxygen reduction) as corrosion-limiting step.

## 2. Materials and Methods

### 2.1. Sample Preparation

Three different biomaterials were used in this work: a low carbon CoCrMo alloy (C: 0.05 %wt, Cr: 28 %wt, Mo: 6 %wt, Co: 65.95 %wt), a titanium Ti64 alloy grade 5 (Ti: bal, Al: 6 %wt, V: 4 %wt) and a pure titanium metal (cp-Ti). The cp-Ti was tested as received and after anodization. The anodization process was carried out in a 0.1 M citric acid solution at an applied voltage of 15 V during 1 min.

Both alloys, CoCrMo and Ti64, were mechanically polished with consecutive 800, 1200, 2400 and 4000 SiC papers. The final surface roughness (Sa) of those materials was 0.2 mm (CoCrMo) and 0.7 mm (Ti64), respectively. The pure titanium and the anodized titanium samples had a surface roughness of 6.5 mm and 6.3 mm, respectively.

Before testing, all samples were ultrasonically washed in an acetone bath followed by an ethanol bath.

### 2.2. Sterilization Processes

In order to analyze the influence of the sterilization process on the corrosion behavior of the biomedical alloys, four consecutive sterilization methods were carried out:

- Ethanol (EtOH): the samples were immersed in a 70% ethanol bath over 20 min.
- Autoclave sterilization (AC): after the EtOH step, samples were introduced in an autoclave at 121 °C at a pressure of 0.1 MPa over 20 min.
- UV-light sterilization (AC-UV): after the EtOH and AC steps, the samples were exposed to UV-Light (260 nm wavelength) over 20 min.
- Gamma radiation sterilization (G): the samples without any previous sterilization were exposed to a source of energy between 25–42 kGy.

### 2.3. Surface Analysis

Before the electrochemical tests, surface analysis of the sterilized CoCr samples was carried out via Auger Electron Spectroscopy (AES) in a PHI 680 Scanning Auger Nanoprobe (Physical Electronics GmbH, Feldkirchen, Germany) using a tilted angle of 30° and an energy of a 10 keV (10 nA) electron beam. Depth profile acquisition was performed by scanning at 1 keV (500 nA) with an Ar+ beam over an area of 2 × 2 mm for the CoCr and titanium samples. For the anodized titanium, higher energy of the ion beam was used, 2 keV (2 µA). The sputter rate of SiO_2_ standards measured under those conditions (1.5 nm/min and 5 nm/min, respectively) was used to convert the sputter time to the approximate sputter depth.

AES depth profiles were smoothened by averaging three acquisition points and then corrected for the influence of the electron escape depth using the same procedure that Mischler et al. used for passive films [16] and further applied for CoCrMo alloys [16]. The peaks considered for analysis and the corresponding values for the electron inelastic mean free path (IMFP) are listed in Table 1.

### 2.4. Electrochemical Measurements

Corrosion behavior was characterized by using a three-electrode electrochemical cell using a calomel (SCE) reference electrode (0.244 V vs. standard hydrogen electrode SHE) and a platinum counter electrode. All potential values in the text refer to that SCE reference electrode. The exposed area of the working electrode was 7 cm^2^. The electrochemical cell was filled with 380 mL of 0.15 M NaCl solution, pH 7, under aerated conditions and connected to an Autolab 301N potentiostat.

The following electrochemical sequence was carried out on the sterilized samples using four electrochemical techniques:

- The Open Circuit Potential (OCP) was continuously measured over 60 min.
- During the OCP measurement, linear polarization measurements were carried out every 10 min from −10 mV to +10 mV with respect to the OCP at a scan rate of 2 mV/s in order to determine the polarization resistance (*R_p_*). *R_p_* is inversely proportional to the corrosion rate.
- Electrochemical impedance spectroscopy (EIS) was carried out at OCP after 60 min of immersion in a frequency range from 10^5^ to 0.01 Hz with an amplitude of 10 mV. This measurement allowed for characterization of the interface biomaterial/electrolyte.
- A cathodic potentiodynamic scan was carried out from OCP towards −200 mV vs. OCP at 2 mV/s. Cathodic kinetic parameters were extracted from those curves.

At least two tests under the same conditions were carried out to check for reproducibility of the data.

Anodic polarization curves from 200 mV below the OCP to 1000 mV at 2 mV/s were also carried out in order to characterize the corrosion behavior of all materials.

## 3. Results

### 3.1. Surface Analysis

**Figure 1:**
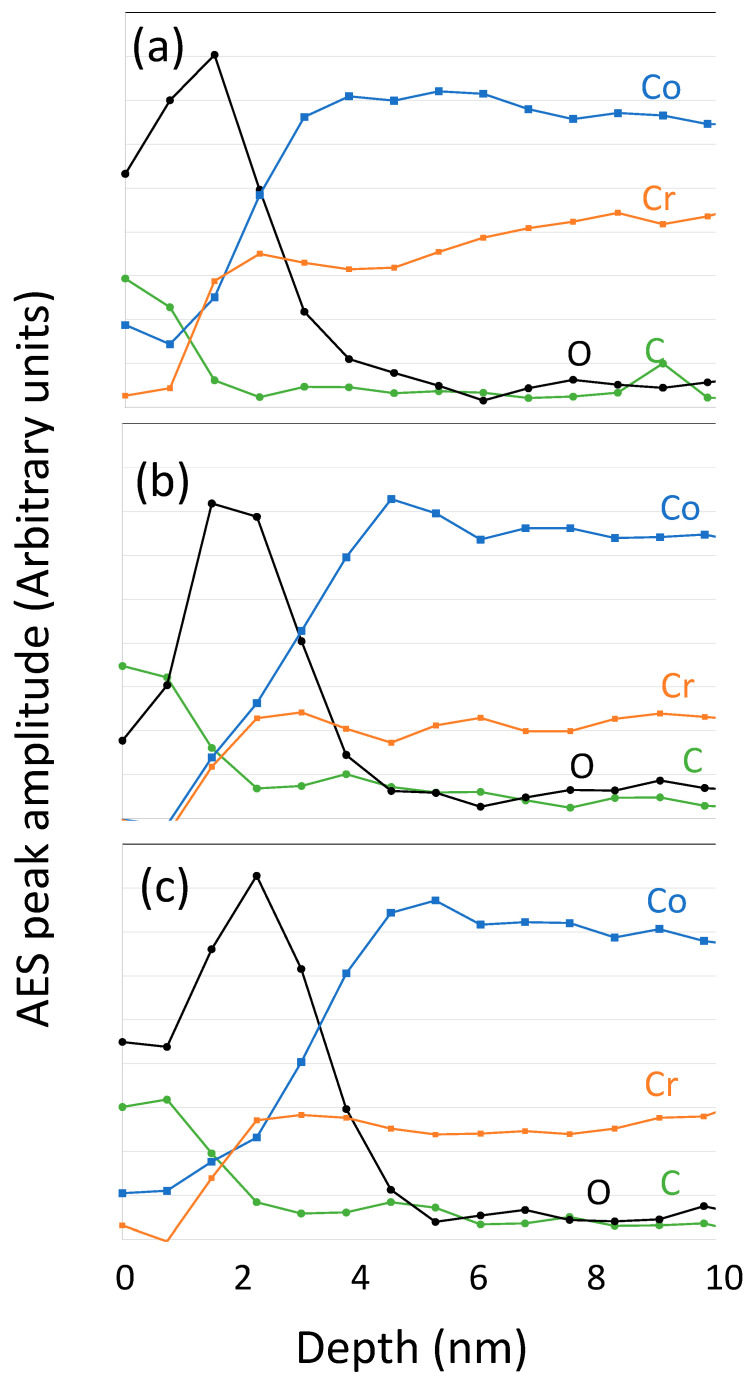
AES sputter depth profiles after correction for the electron escape depth measured on CoCrMo samples sterilized by (**a**) EtOH, (**b**) AC and (**c**) AC-UV.

Figure 1 shows typical AES profiles of the CoCrMo samples subjected to different sterilization methods, considering the characteristic peaks of C, O, Co and Cr. Although Mo was also identified in the spectra measured before each depth profile, the signals were very weak and they have not been plotted in the figure. The intensity of the oxygen signal passes through a maximum with the sputter depth and starts decreasing again around 2 and 2.5 nm. The thicknesses of the passive layers can be determined by taking the depths at which the oxygen signal is at 50% of its maximum amplitude, which correspond to 3, 4.5 and 4.8 nm depending on the sterilization process, EtOH, AC or ACUV, respectively.

The oxide film thickness and composition for the titanium and the anodized titanium was slightly influenced by the sterilization process. In all cases, the film was composed of TiO_2_. In the titanium sample, an oxide film of around 30 nm was observed, while on the anodized titanium, an oxide layer was formed at around 300 nm. Only in the case of sterilization with EtOH was the passive film thinner when compared to the other sterilization methods.

### 3.2. Electrochemical Measurements

**Figure 2:**
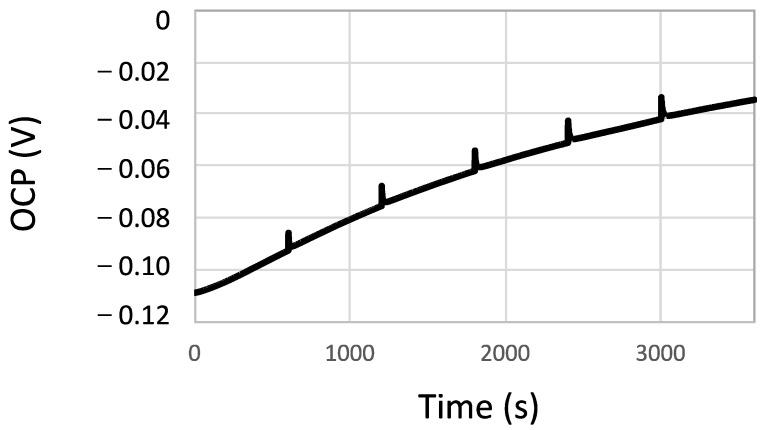
OCP evolution with time of the CoCr alloy sterilized by autoclaving in the NaCl solution.

The Open Circuit Potential (OCP) of the tested samples was continuously monitored with time, and every 10 min, polarization resistance was measured based on a short scan of ±10 mV from the OCP. Figure 2 shows a typical example of the OCP evolution with time of the CoCr alloy sterilized by autoclaving. The OCP transients observed every 10 min corresponded to the moments at which the *R_p_* was measured. A moderate increase in OCP with time, typically from passive materials, was observed in all samples.

**Figure 3:**
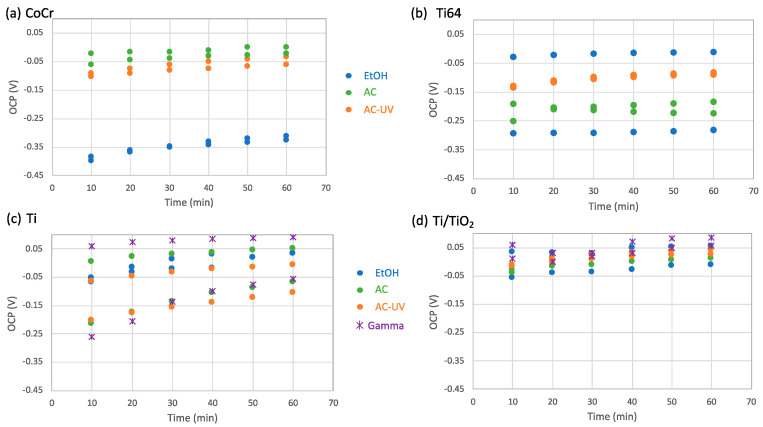
Evolution with time of OCP values obtained every 10 min of immersion for the (**a**) CoCr, (**b**) Ti64, (**c**) Ti and (**d**) Ti/TiO_2_ in the NaCl solution.

Figure 3 shows the OCP values determined every 10 min before the measurement of the *R_p_* and their evolution with time for the different materials sterilized by different processes. OCP values of the CoCr alloy are very reproducible and slightly increase with immersion time in the saline solution (Figure 3a). The main effect on OCP was observed with autoclave sterilization, which shifts the OCP towards higher values compared to those with the EtOH process. Exposure to UV-light after AC sterilization also generates a slight increase in OCP. In the titanium samples, no general effect of sterilization was observed. The OCPs of the Ti64 alloy, in Figure 3b, reached values between 0 and −0.3 V after sterilization in EtOH. Autoclaving reduces the scatter of the OCP values, with all of them being around −0.17 V, and exposure to the UV-light slightly shifted those OCPs towards higher values, around −0.07, at the end of the OCP tests. In the AC-UV sterilization process, the scatter of the OCP values was very low, at ± 20 mV. On the contrary, the lowest scatter in the Ti samples was obtained after sterilization in EtOH, and in this case, no clear effect of the sterilization process on OCP was observed (Figure 3c). Finally, when Ti was anodized, all OCP values remained very constant during the whole test and lied between a range of 50 mV, independently of the sterilization process (Figure 3d).

**Figure 4:**
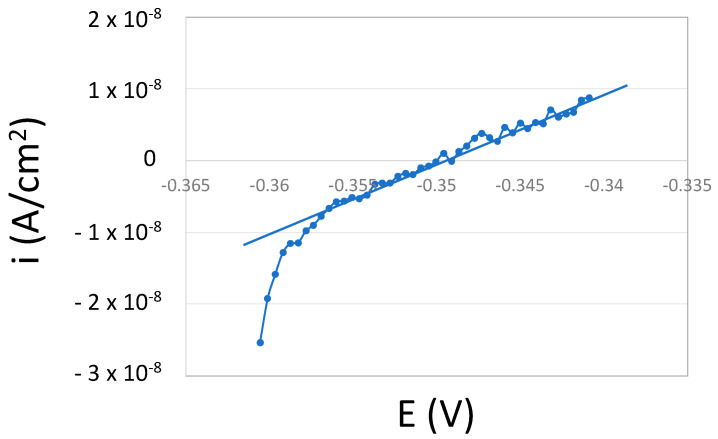
*R_p_* measurements of CoCrMo sterilized by EtOH after 30 min of immersion in NaCl. The dots correspond to the experimental data and the line, the slope of the linear part, which is proportional to the polarization resistance (*R_p_*).

The polarization resistance, determined as the slope of the linear regression of the potential versus the current around the potential of zero current, was obtained every 10 min. Figure 4 shows a typical example of a polarization resistance measurement. The deviations from linearity obtained at cathodic potentials are probably due to capacitive effects caused by the charging of the double-layer and the passive film.

**Figure 5:**
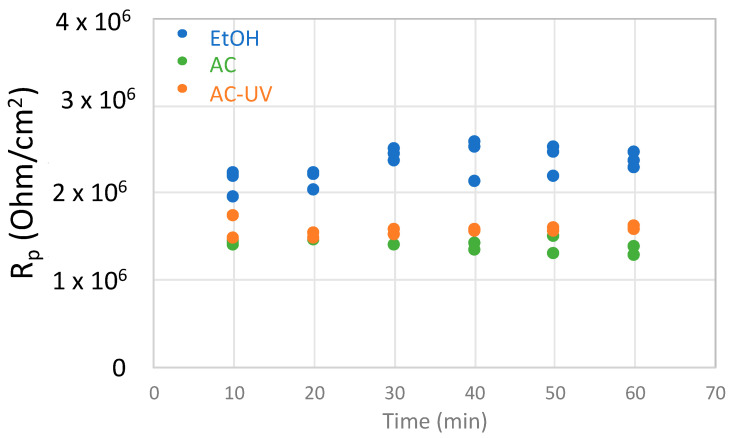
Evolution with time of *R_p_* values determined every 10 min of immersion in the NaCl solution for the Ti64 alloy sterilized by EtOH, AC and AC-UV.

Figure 5 shows the evolution with time of the extracted *R_p_* values every 10 min for the Ti64 sample sterilized using different procedures. In general, all materials show very reproducible polarization resistance values, shifting towards slightly higher values with time.

**Figure 6:**
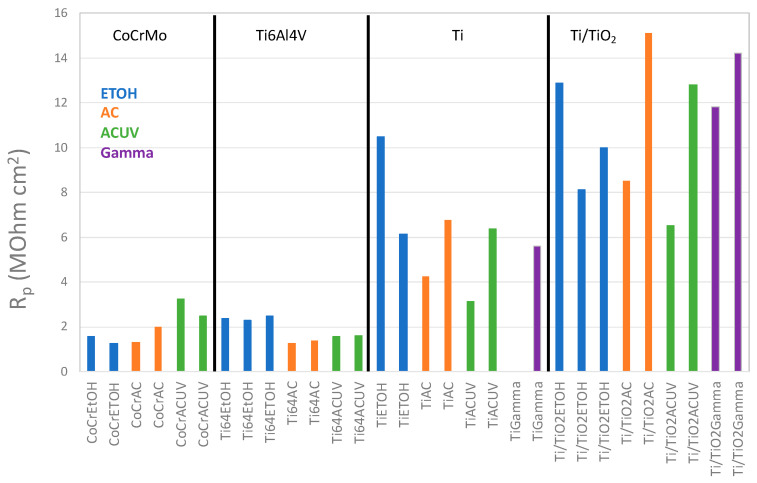
*R_p_* values determined after 60 min of immersion in the NaCl solution of all tested biomaterials sterilized by EtOH, AC and AC-UV.

For comparison purposes, Figure 6 shows the summary of the *R_p_* values obtained after 60 min of immersion in the NaCl solution for all tested samples. The highest *R_p_* values were obtained for the anodized titanium samples followed by the pure titanium ones. The *R_p_* values of the CoCr and the Ti64 alloys were modified by the sterilization process. Autoclaving and UV-light exposure increased the *R_p_* of the CoCr, while they decreased the *R_p_* value for the Ti64.

**Figure 7:**
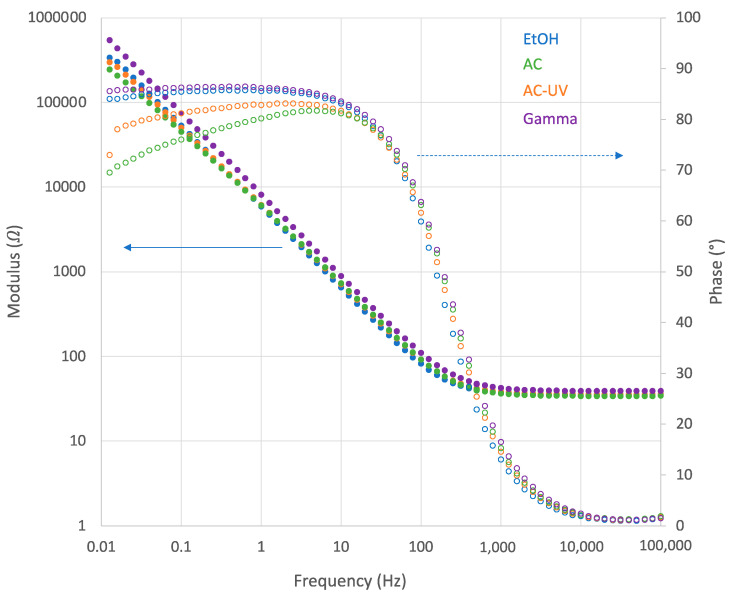
Bode plots of the pure Ti sterilized by EtOH, AC, AC-UV and gamma radiation after 60 min at OCP in the NaCl solution.

Electrochemical impedance spectroscopy was carried out at the OCP measured after 60 min of immersion. Figure 7 shows some examples of Bode plots, the modulus |Z| (in logarithmic scale) and the phase shift (both on the *y*-axis) as a function of the logarithm of the frequency f, of the pure Ti obtained at the OCP established after 60 min of immersion in NaCl after being sterilized using different processes. It constitutes the typical spectra of passive materials with high impedance at low frequencies and a broad phase angle maximum. Similar spectra were obtained for all tested samples.

In those spectra, the highest frequency region shows a constant value for log|Z| with a phase shift close to 0°. This resistive behavior of the impedance corresponds to the solution resistance *R_s_*, i.e., the solution resistance between the working and the reference electrode. These *R_s_* values lied around 35 Ω in all tests, in good agreement with the good electrical conductivity of saline solutions.

**Figure 8:**
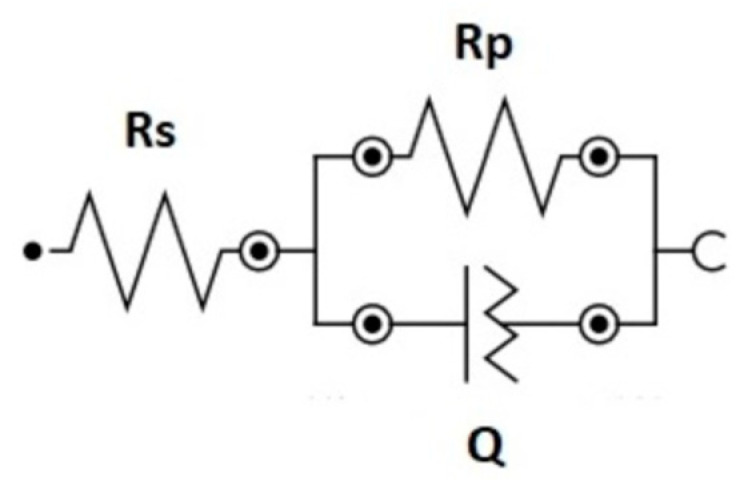
Randles circuit composed of a solution resistance (*R_s_*) coupled to a parallel circuit constituted by the polarization resistance (*R_p_*) and a constant-phase element (*Q*).

At the lowest frequencies, no plateau of log|Z| values was reached. This indicates that the time constant of the electrochemical process is low, and thus, the polarization resistance could not be extracted from the EIS results. Therefore, the polarization resistances were obtained from the DC measurements at OCP and the capacitance of the interface calculated using a simple electrical model, shown in Figure 8. A constant *R_s_* value of 35 Ω was considered.

This equivalent electrical circuit (EEC) consists of the electrolyte resistance *R_s_* in series with the parallel combination of the oxide capacitance *C_ox_* and the impedance of the faradaic reaction, the polarization resistance. In order to consider different surface physical phenomena, such as surface heterogeneity, which results from surface roughness, impurities, dislocations or grain boundaries [17], instead of an ideal capacitor, a constant phase element (*Q*) is used. The relationship between *Q* and *C_ox_* is given by Equation (1), proposed by Brug et al. [18]:

(1) $$C_{ox}={\left(\frac{Q}{R_{s}^{-1}+R_{p}^{-1}}\right)}^{\frac{1}{n}}$$

This approach has been already used and validated by other authors for determining the capacitance values of different passive materials [19,20,21].

Table 2 summarizes the fitted values of the capacitance and the OCP values at which the EIS was carried out for all samples. Two repetitions under the same experimental conditions are shown.

**Figure 9:**
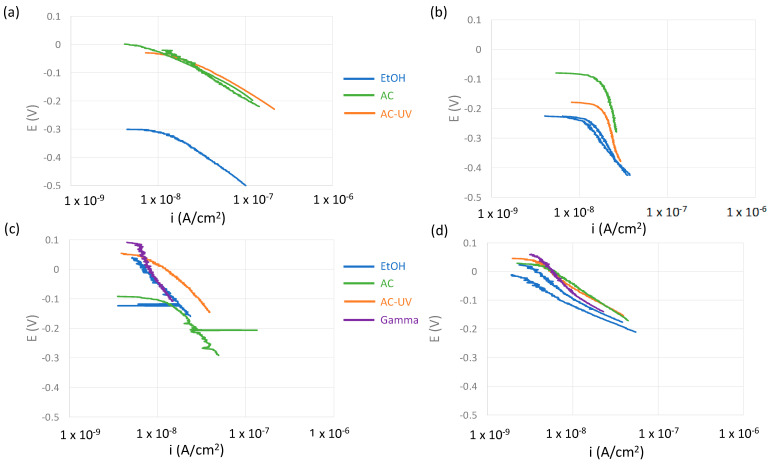
Cathodic polarization curves from OCP of (**a**) CoCr, (**b**) Ti64, (**c**) Ti and (**d**) Ti/TiO_2_ in the NaCl solution.

After the EIS measurement, cathodic polarization from the OCP towards lower potential values was carried out. Figure 9 shows those cathodic polarization curves of the studied biomaterials sterilized using different processes.

In the CoCr alloy (Figure 9a), independent of the sterilization process, the relationship between potential and the logarithm of the current density shows a linear relationship, which means that the cathodic reaction (oxygen reduction) is not controlled by mass transport, but by charge transfer. A single cathodic slope was determined to lie between 70 and 90 mV. Similar cathodic slopes were found for the Ti/TiO_2_ sample (Figure 9d) sterilized using different processes at low overpotentials. However, the cathodic behavior of Ti/TiO_2_ shows a change in the slope towards lower values (around 50 mV) when cathodically polarizing the metal below 0.1 V with respect to the initial OCP. Similar behavior was found in the titanium alloy and pure titanium depending on the sterilization process. The values of the cathodic slopes of the studied samples are summarized in Table 3.

## 4. Discussion

### 4.1. Oxygen Reduction Kinetics

During the sterilization procedure, a segregation process might occur or the surface composition could be altered because of the addition or elimination of a contaminant. Besides this, the thickness of the oxide layer might also be affected by the steps of the sterilization procedure. As a result of the sterilization steps, changes in the surface composition and thickness of the oxide layer may be expected. Consequently, the sterilization would not only be a microbe-killing process but could also produce a surface with different characteristics. Therefore, different interaction effects between the implant surface and body tissue (with the subsequent effect on the implantation process) could be expected depending on the material.

Electrochemical kinetic parameters (cathodic Tafel slope, b_c_, corrosion current density, i_corr_, and exchange current density, i_0_, for oxygen reduction) were extracted from the polarization curves shown in Figure 9, and they are summarized in Table 3. The corrosion rates, expressed as corrosion current densities, i_corr_, were calculated from the polarization resistance values *R_p_* (Figure 4) and the cathodic Tafel slope, simplifying the Butler–Volmer equation of a mixed electrode when the anodic Tafel slope (b_a_) is much higher than the cathodic one (i.e., passive materials).

i_corr_ = β_c_ β_a_ (β_c_ + β_a_)^−1^ R_p_^−1^ ≈ β_c_
*R_p_*^−1^

The cathodic reaction at the measured potentials corresponds to the oxygen reduction reaction (O_2_ (g) + 2H_2_O + 4e^−^ ⇌ 4OH^−^ (aq)) and was obtained from the Tafel plots shown in Figure 9 and by extrapolating the current density to E_O2/OH−_ = 0.57 V_SCE_ (equilibrium potential for oxygen reduction at pH 7).

### 4.2. Effect of Sterilization on the Electrochemical Behavior of the CoCrMo Biomedical Alloy

In CoCrMo, successive sterilization steps increases the corrosion resistance (up to an inhibition effect of 66% in ACUV with respect to EtOH). 

**Figure 10:**
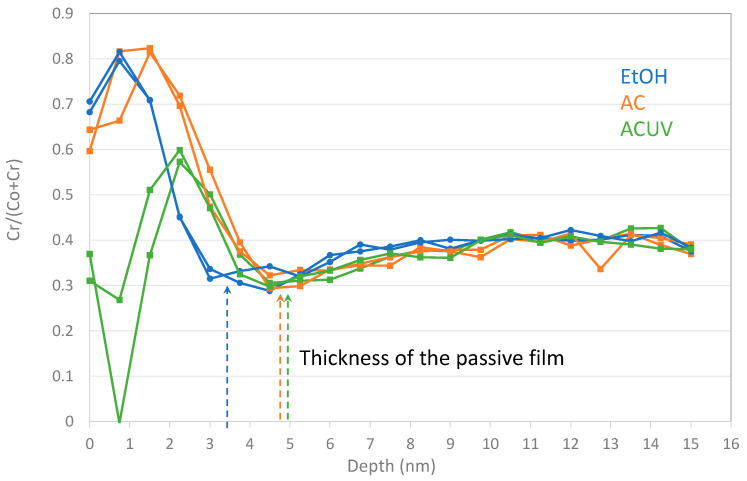
Cr ratio as a function of depth in the CoCrMo alloy.

The effect of AC and ACUV sterilization on the CoCrMo are related to a change in the surface chemistry. Figure 10 shows the Cr ratio with respect to the total Co and Cr concentration as a function of the sputter depth. It is possible to observe an enrichment in Cr in the passive film. This suggests a preferential Co dissolution during EtOH sterilization. On the other hand, AC provokes an increase in the thickness of the passive film. This chemical effect of sterilization on the surface is in good agreement with the OCP shifts towards higher values in the EtOH < AC < ACUV CoCrMo samples (Figure 3). In the figure, clearly, the main effect on the OCP was caused by the autoclaving. The autoclave process, a heat treatment at 121 °C, the oxide growth rate may increase with an increase in the diffusion coefficients caused by the temperature increase. As shown in the AES depth profiles of Figure 1, the thicknesses of the passive layers increase with the sterilization method from 3 nm with EtOH to 4.5 nm with AC and 4.8 nm with ACUV. Moreover, the presence of impurities might affect the oxide growth rate by enhancing mass transport and/or the adsorption and dissociation of oxygen at the surface.

**Figure 11:**
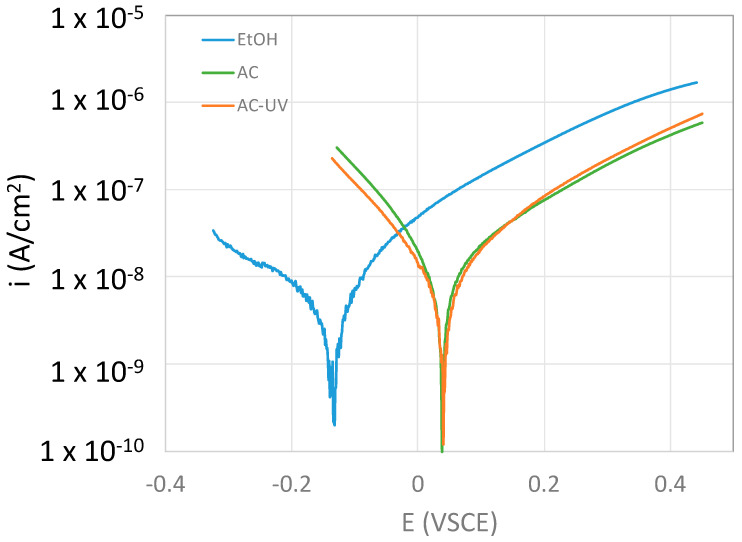
Anodic polarization curves (potential scan from cathodic towards anodic potentials at 2 mV/s) of the CoCrMo alloy after different sterilization methods in the NaCl solution.

**Figure 12:**
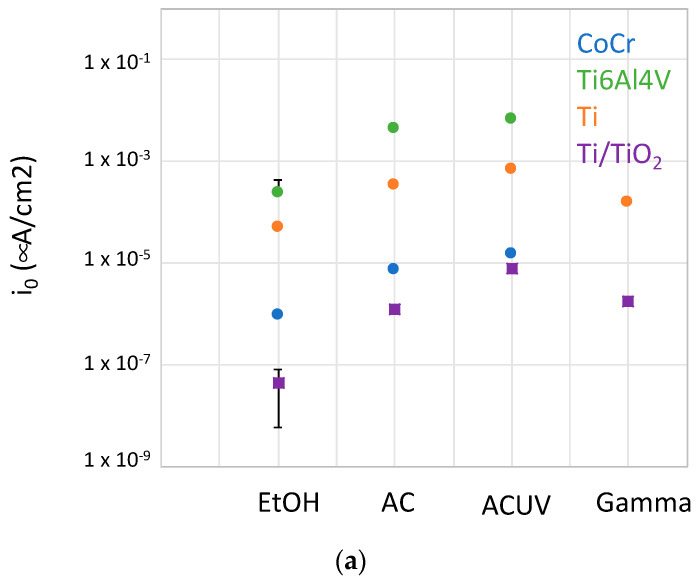

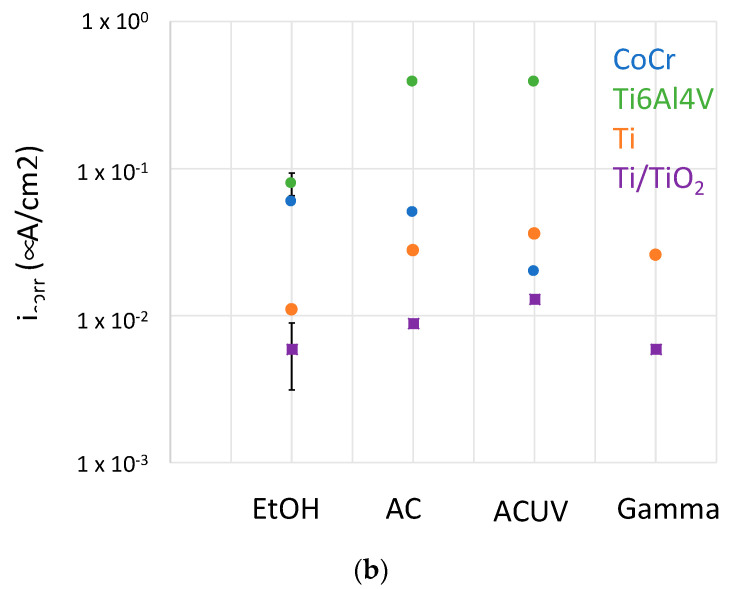
(**a**) Exchange current densities for oxygen reduction and (**b**) corrosion current density on the different sterilized samples.

Considering that the anodic behavior of the CoCrMo alloy is independent of the sterilization process (Figure 11), its electrochemical behavior can be rationalized through the kinetics of the reduction reaction. The cathodic kinetics for oxygen reduction on the CoCrMo surface are kinetically controlled according to the polarization curves shown in Figure 9. Indeed, the exchange current densities for this cathodic reaction on the sterilized CoCrMo surfaces are very low because they are dramatically inhibited by the passive film. This inhibition is dependent on the sterilization process, and i_0_ is lower in EtOH < AC < ACUV (Figure 12a). The cathodic Tafel slope is very similar in all cases, around 0.080 V/decade, so the variation of i_0_ is caused by the lower OCP of the CoCrMo after EtOH sterilization, as a consequence of the different surface chemistries (Figure 10). However, the corrosion current densities of the CoCrMo alloy increase with the increase in sterilization steps, EtOH > AC > ACUV (Figure 12b). In any case, corrosion current densities are in the range of tenths of nA/cm2, typical values for passive materials. Therefore, the electrochemical behavior of this alloy is influenced not only by the cathodic kinetics but also by their passivity.

### 4.3. Effect of Sterilization on the Electrochemical Behavior of Titanium and Titanium Alloy Biomedical Alloy

Successive sterilization steps carried out on titanium and titanium alloys decrease the corrosion resistance with respect to EtOH. The effect of AC and ACUV is specially marked in the case of the Ti64 alloy, for which the corrosion rate increases one order of magnitude compared to the corrosion rate after EtOH sterilization. This corresponds well with the increase in the i0 for oxygen reduction (Figure 12a), thus suggesting cathodic control of the corrosion process for titanium and its alloys. Indeed, the sterilization process can modify up to two orders of magnitude the exchange current density for oxygen reduction. This effect is more pronounced in the case of the anodized titanium.

Autoclaving significantly increases the oxygen reduction kinetics of titanium and its alloys. The possible generation of defects on the titanium passive film after the heat treatment produced during autoclaving could be the responsible for this effect, while UV only modifies the anodized titanium to the same extent as AC. This is not surprising since UV irradiation was reported to create oxygen vacancies due to the formation of two coordinated bridging sites, which converts the corresponding Ti^4+^ sites to Ti^3+^ sites that enhance the amount of absorbed dissociated water and increase the surface energy [22]. On the thin spontaneously formed passive layers (Ti and Ti64), this effect is less marked than in the much thicker oxide formed on the anodized titanium.

## 5. Conclusions

The influence of different sterilization process (ethanol, autoclave, UV-light and gamma radiation) on the corrosion resistance of CoCrMo and titanium biomedical alloys has been assessed and the following outcomes have been obtained:

- For CoCrMo alloys, sterilization by AC and ACUV significantly modified the surface composition (thicker and chromium rich passive film) and improved the corrosion resistance compared to simple sterilization in EtOH. The modified surface composition was found to inhibit the oxygen reduction reaction, thus reducing the corrosion rate.
- For titanium and titanium alloys, the sterilization has a negative impact on the corrosion resistance. Indeed, the corrosion behavior was found to be controlled by the oxygen reduction reaction rate, which is enhanced by the AC and ACUV sterilization methods. On the contrary, gamma irradiation slightly increases the corrosion resistance of the investigated titanium and titanium alloy.
- Sterilization methods potentially lead to different reactivities of biomedical alloys and therefore their further biological and clinical performance.
- Further analysis in more complex simulated body fluids (i.e., containing proteins and other organic molecules, controlled oxygen content) together with in vivo measurements could be carried out to optimize the sterilization procedures allowing for minimization of metal ion release in patients.

## Figures and Tables

**Table 1:** AES peaks considered for depth profiling.

Element	Peak	Kinetic Energy (eV)	IMFP (nm)
C	KLL	273	0.7
O	KLL	503	1.0
Cr	LMM	528	1.1
Co	LMM	778	1.4
Ti	LMM	421	1.0

**Table 2:** OCP values at which EIS was carried out and capacitance of the interface (*C_ox_*) determined by fitting the EIS data to the randles EEC of Figure 8 for the different samples.

Sample	Sterilization	OCP (V)	*C_ox_* (μF/cm^2^)
CoCr	EtOH	−0.321	4
		−0.305	4
	AC	−0.029	1.5
		−0.058	0.9
	AC-UV	0	0.8
		−0.235	1.5
Ti64	EtOH	−0.278	1.8
		−0.009	1.8
		−0.225	2.2
	AC	−0.178	1.5
		−0.146	1.5
	AC-UV	−0.079	2
		−0.085	1.8
Ti	ETOH	0.040	3
		0.057	3.1
	AC	0.055	2.5
		−0.059	2.5
	AC-UV	−0.091	2.4
		−0.008	2.7
	Gamma	0.090	2.1
		−0.040	2.1
Ti/TiO_2_	ETOH	−0.011	1
		0.055	1.2
		0.024	1.1
	AC	0.046	1.1
		0.017	1.1
	AC-UV	0.030	1.2
		0.045	0.9
	Gamma	0.060	0.7
		0.090	0.7

**Table 3:** Kinetic parameters calculated from the potentiodynamic curves, in Figure 9, for the different samples in the NaCl solution.

Sample	βc (V/Decade)	i_corr_ (nA/cm^2^)	i_0_ (μA/cm^2^)
CoCrEtOH	0.092	59	9.4 × 10^−7^
CoCrAC	0.077	50	7.1 × 10^−6^
CoCrACUV	0.086	20	1.4 × 10^−5^
CoCrACUV	0.080	25	1.7 × 10^−5^
Ti64EtOH	0.167/0.122	70	6.3 × 10^−5^
Ti64EtOH	0.225/0.163	90	3.8 × 10^−4^
Ti64AC	0.498/0.291	389	4.1 × 10^−3^
Ti64ACUV	0.610	386	6.5 × 10^−3^
TiETOH	0.116	11	4.9 × 10^−5^
TiAC	0.121	28	3.2 × 10^−4^
TiACUV	0.114	36	6.8 × 10^−4^
TiGamma	0.100/0.146	26	1.5 × 10^−4^
Ti/TiO_2_EtOH	0.075/0.052	4	1.7 × 10^−8^
Ti/TiO_2_EtOH	0.090/0.056	8	6.9 × 10^−8^
Ti/TiO_2_AC	0.100/0.070	9	1.2 × 10^−6^
Ti/TiO_2_ACUV	0.086	13	7.9 × 10^−6^
Ti/TiO_2_Gamma	0.123/0.075	6	1.7 × 10^−6^

## Data Availability

Data can be obtained upon reasonable request to the main author (AIM).
